# Detecting, Quantifying, and Isolating Monkeypox Virus in Suspected Cases, Spain

**DOI:** 10.3201/eid2907.221229

**Published:** 2023-07

**Authors:** Marta E. Álvarez Argüelles, Zulema Pérez Martínez, Susana Rojo Alba, Jose María González Alba, Ana María Fernandez-Verdugo, Isabel Costales González, Garbriel Martín Rodríguez, Jose Antonio Boga Riveiro, Mario Margolles Martins, Santiago Melón García

**Affiliations:** Hospital Universitario Central de Asturias, Oviedo, Spain (M.E. Álvarez Argüelles, Z. Pérez Martínez, S. Rojo Alba, J.M. González Alba, A.M. Fernandez-Verdugo, I. Costales González, G.M. Rodríguez, J.A. Boga Riveiro, S. Melón García);; Epidemiological Surveillance Department, Oviedo (M. Margolles Martins)

**Keywords:** mpox, monkeypox virus, orthopoxvirus, real-time PCR, cell culture, sexually transmitted infections, Spain, viruses

## Abstract

When a monkeypox virus outbreak began in several parts of the world in May 2022, timely and accurate diagnosis became mandatory. In our laboratory, a real-time quantitative PCR was designed and evaluated in several patient samples and compared with isolation results. Genomic viral load was related to virus viability.

After smallpox was eradicated worldwide in 1980 and routine smallpox vaccination subsequently ceased, monkeypox virus (MPXV) emerged as the most consequential orthopoxvirus for public health (*1*). The genetic clades of MPXV are clade I (formerly the Congo Basin [Central African] clade), which is associated with higher virulence and greater mortality rate, and clade II (formerly the West African clade) (*1*,*2*).

In May 2022, the United Kingdom reported an outbreak of mpox (formerly monkeypox) that subsequently spread globally (*3*); Spain was one of the most affected countries (*4*). The rapid increase in mpox cases has challenged clinical laboratories to understand the spread and transmission of MPXV. We sought to detect and isolate MPXV in persons at risk for infection by genomic amplification using a real-time quantitative PCR (qPCR) and isolation in culture cell.

This study did not require research ethics committee approval because it describes analyses that were completed at the public laboratory as part of routine clinical testing and surveillance during the mpox outbreak in Asturias in the northwest of Spain. Therefore, this study was considered public health practice and was exempt from this requirement.

## The Study

During May 24–July 15, 2022, a total of 66 samples (42 lesion swabs, 20 respiratory samples, and 4 blood samples) belonging to 41 adults (mean age 35.61 ± 11.25, range 17–60 years) and collected within 72 hours of illness onset were submitted in accordance with laboratory requirements (Table 1). All patients were located in a 30-km area around Oviedo, the capital of Asturias. In addition to MPXV, we also tested for herpes simplex virus, varicella zoster virus, enterovirus, human herpesvirus 8, molluscum contagiosum virus, and human papilloma virus, according to clinical manifestations. Samples were processed following laboratory protocols for nucleic acid detection, and 21 were inoculated onto monolayer conventional cell culture (MRC-5 cell, Vero-E6, and A549 and LLC-MK2 cells subcultures).

**Table 1:** Characteristics, sample type, and clinical features for patients in study of MPXV detection and isolation, Spain*

Patient no.	Date	Age, y/sex	HIV serostatus	MSM/SP†	Sample type	Clinical features
1	2022 May 24	54/F	–	No	Lesion swab	Rash, fever
2	2022 May 25	17/M	–	NA	Pharyngeal and oral swab	Acute pharyngitis
3	2022 Jun 15	34/M	+	Yes	Lesion swab	Genital lesions
4	2022 Jun 15	34/M	–	Yes	Lesion swab	Lesions, fever, adenopathies
5	2022 Jun 15	49/M	+	Yes	Nasopharyngeal and lesion swab	Perioral lesions
6‡	2022 Jun 17	39/M	+	Yes	Nasopharyngeal and lesion swab	Lip lesion, fever
7	2022 Jun 18 and 21	45/M	+	Yes	Pharyngeal and lesion swabs	Perianal and pharyngeal discomfort, vesicular exanthema
8	2022 Jun 20	25/F	–	No	Nasopharyngeal and lesion swab	Lesion
9	2022 Jun 20	49/M	–	Yes	Lesion swab	Lesion
10	2022 Jun 20 and 24	41/M	–	Yes	Nasopharyngeal and lesion swab	Febrile, pubic lesion
11	2022 Jun 21	45/M	–	Yes	Lesion swab	Folliculitis in the context of scabies
12	2022 Jun 24	43/M	–	Yes	Lesion swab	Odynophagia, folliculitis, genital lesion, fever
13	2022 Jun 30	37/M	–	Yes	Lesion swab	Cutaneus and genital lesions, adenopathies
14	2022 Jul 1	35/M	+	Yes	Nasopharyngeal, lesion swab, blood	Cutaneus and genital lesions, fever, adenopathies
15	2022 Jul 1	29/M	+	Yes	Nasopharyngeal and lesion swab	Vesicular lesions on trunk, palms, and genitals, fever
16	2022 Jul 2	35/M	+	No/Yes	Lesion swab	Odynophagia/cutaneus and genital lesions, fever, adenopathies
17	2022 Jul 2	22/M	–	No/Yes	Lesion swab	Genital lesions
18	2022 Jul 3	47/M	+	No	Lesion swab	Cutaneus and genital lesions
19	2022 Jul 4	30/M	–	Yes	Nasopharyngeal and lesion swab	Lesions, fever
20‡§	2022 Jul 4	26/M	–	Yes	Lesion and rectal swabs, blood	Lesions, fever
21	2022 Jul 5	22/M	–	NA	Lesion swab	Itchy lesions
22	2022 Jul 5	41/M	+	NA	Lesion swab	Lesions¶
23	2022 Jul 5	48/M	–	NA	Lesion swab	Lesions¶
24	2022 Jul 5	30/M	+	NA	Nasopharyngeal and lesion swab	Lesions¶
25	2022 Jul 6	26/M	–	NA	Nasopharyngeal and lesion swab	Lesions¶
26	2022 Jul 7	29/M	–	Yes	Lesion swab	Lesions¶
27	2022 Jul 8	28/M	–	NA	Lesion swab	Lesions¶
28	2022 Jul 8	60/M	–	NA	Pharyngeal and lesion swab	Lesions¶
29	2022 Jul 8	22/M	+	Yes	Pharyngeal and lesion swab, blood	Cutaneus and genital lesions
30	2022 Jul 11	54/M	NA	NA	Lesion swab	Lesions¶
31	2022 Jul 11	47/M	–	Yes	Pharyngeal and lesion swab	Lesions, adenopathies
32	2022 Jul 12	30/M	NA	NA	Lesion swab	Lesions¶
33	2022 Jul 12	32/F	NA	NA	Lesion swab	Lesions¶
34	2022 Jul 12	34/F	–	No	Pharyngeal and lesion swab	Necrotic lesions on forehead
35	2022 Jul 13	19/F	NA	NA	Pharyngeal and lesion swab, blood	Umbilical lesions
36	2022 Jul 13	56/M	NA	NA	Pharyngeal and lesion swab	Lesions¶
37	2022 Jul 13	21/M	–	No	Lesion swab	Lesions¶
38	2022 Jul 13	19/F	–	No	Lesion swab	Lesions¶
39	2022 Jul 14	34/F	–	No	Pharyngeal and lesion swab	Back rash in different stages of evolution
40	2022 Jul 15	42/M	NA	NA	Lesion swab	Lesions¶
41	2022 Jul 15	30/M	+	Yes	Pharyngeal and lesion swab	Lesions, adenopathies

*Gray shading indicates MPXV-infected patients. MPXV, monkeypox virus; MSM, men who have sex with men; NA, not available; SP, high-risk sexual practices; +, positive; –, negative. †No further information was available regarding attending clinicians’ criteria for high-risk sexual practices. ‡Inpatient. §Renal transplant patient. ¶No further clinical information available.

We extracted nucleic acids by using the automated nucleic acid purifier Magnapure 96 (Roche Diagnostics, https://www.roche.com). We performed orthopoxvirus group PCR (*5*) and specific MPXV in-house real-time qPCR.

For qPCR, we amplified 5 µL of extracted nucleic acids in a final volume of 10 µL, including the Brillant III Ultra-fast QPCR Master MIX (Agilent Technologies, https://www.agilent.com), 1,000 nm of each primer (MPXV-S, TGTTGACGCACCAGCGTCT; MPXV-A, AACAGTGGACCCTTGATGACTGT), and 200 nm of FAM-labeled MGB probe (CAATCCATGGTATTCGA; ABI, CA). We performed qPCR as follows: 95° for 7 min, 45 cycles of 95° for 5 min and 60° for 33 min. In addition, we quantified the human β-globin gene in each sample to evaluate sample quality and to calculate normalized viral load in log_10_ copies per 10^3^ cells (*6*).

We detected MPXV in 23 (56.09%) of the 41 patients studied. All were men; the mean age was 38.13 + 9.42 years (range 22–56 years). At least 15 stated they had sex with men, and 12 were HIV-positive. We detected herpes simplex virus 1 in 2 persons (1 of whom was co-infected with MPXV) and varicella zoster virus 1 person. MPXV was detected in 25 (96.15%) lesion swab samples with a viral load of 7.58 + 2.03 (range 3.84–11.71 [95% CI 6.44–8.19]) and in 8 (66.66%) respiratory swab specimens with a viral load of 5.04 + 1.01 (range 3.5–6.23 [95% CI 4.192–5.88]; p = 0.0041).

**Figure 1:**
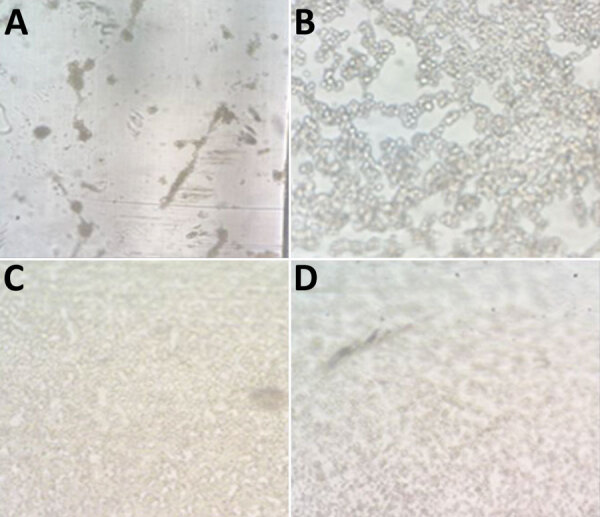
Cytopathic effect in monkeypox virus–infected cells from patients in Asturias, Spain. A) MRC-5; B) Vero E6; C) A549; D) LLC-MK2. Original magnification ×10.

We isolated virus in 13 (81.25%) patients out of 16 infected persons inoculated. From those persons, we recovered MPXV in 17 (80%) of 21 samples assayed: 4 (80%) respiratory swab samples (3 from Vero-E6 and 1 from MRC-5 cells) and 13 (81.25%) lesion swab samples (13 from Vero-E6 cells and 11 from MRC-5 cells) (p = 0.68) (Table 2). In 6 subcultures in A549 cells and in LLC-MK2 cells, cytopathic effect was observed and confirmed by PCR (Figure 1).

**Table 2:** Results of days of isolation and average cycle threshold values of samples according to results of culture in study of MPXV detection and isolation, Spain*

Cell	No. positive cultures	Mean days of positivity +SD (range) [95% CI]	Mean Ct of positive cultures +SD (range) [95% CI]	No. negative cultures	Mean Ct of negative cultures +SD (range) [95% CI]	p value
MRC-5 cells	12	5.8 +2.4 (3–10)† [4.2–7.4]	22.4 +6.4 (15–34) [18.3–26.5]	9	29.5 +3.1 (25–34) [27.1–31]	0.0068
VeroE6 cells	16	3.7 +1.3 (3–7)† [2.9–4.5]	23.4 +5.8 (15–34) [20.3–26.4]	5	32.02 +1.1 (31–34) [30.8–33.5]	0.0035
Both lines	17	3.7 +1.3 (3–7) [2.9–4.5]	23.9 +5.9 (15–34) [20.8–26.9]	4	32.25 +1.25 (31–34) [30.2–34.2]	0.0099

*Ct, cycle threshold; MPXV, monkeypox virus. †p = 0.012.

**Figure 2:**
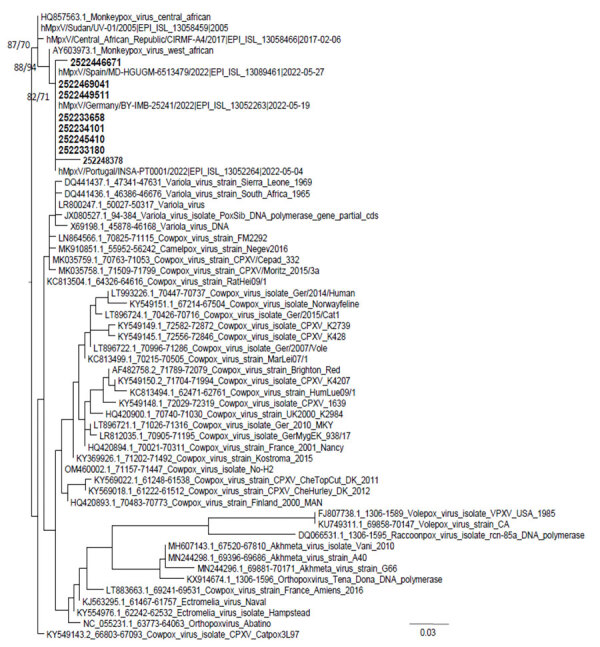
Phylogenetic relationships of monkeypox virus strains from patients in Asturias, Spain (bold), and reference strains from GenBank and GISAID (https://www.gisaid.org). Alignment has 57 sequences with 262 nucleotides. Numbers in nodes are SH-aLRT support (%)/ultrafast bootstrap support (%). Scale bar indicates number of base substitutions per site.

We characterized the virus using Sanger sequencing method, and we purified then sequenced the PCR product by using BigDye Terminator v1.1 Cycle Sequencing Kit with an ABI PRISM 3700 DNA analyzer (both ThermoFisher Scientific, https://www.thermofisher.com). We analyzed the sequences subsequently obtained by using IQ-TREE multicore version 2.1.3 (http://www.iqtree.org). We performed tree reconstruction using best-fit model chosen according to Bayesian information criterion. We tested tree branches by the SH-like aLRT method with 1,000 replicates, generating 1,000 samples for ultrafast bootstrap (Figure 2).

## Conclusions

In MPXV infection, human-to-human transmission can result from close contact with respiratory secretions, skin lesions of an infected person, or recently contaminated objects (*7*). In this study, all infected patients were men who had skin lesions. In <50% of cases, patients were experiencing fever, adenopathy, or malaise. Fifteen patients stated they had sex with men or engaged in high-risk sexual practices. In this study, 12 HIV-positive patients were infected with MPXV but did not have higher viral load (*4*). Only 2 patients were hospitalized. One patient had fever and severe lymphocytosis. The other was a renal transplant patient hospitalized mainly because of his immunosuppressed state. In that patient, the virus was detected for >1 week. In any case, all the patients had a positive outcome.

All viruses were characterized as clade II, similar to findings from other cases in Spain, suggesting the same focus of infection (S. Buenestado et al., unpub. data, https://virological.org/t/updatetwo-draft-genomes-from-madrid-spain-of-the-monkeypoxvirus-2022-outbreak/848). As expected, the disease followed a self-limited course, and no patients experienced severe complications. DNA sequencing also makes it possible to interpret transmission episodes and confirm the existence of endemic variants (*8*,*9*). Community transmission data were available for 10 cases. Only 2 were considered secondary cases, indicating that transmission in that environment at that point was not common but had started and could spread.

When possible, blood and pharyngeal or nasopharyngeal swabs were collected, per World Health Organization recommendations (*7*,*10*). Blood samples were only collected from 3 patients, and virus was detected in 2 of them at <4 log_10_ copies/mL. Viremia occurs very early in the course of infection and usually contains a lower viral load than lesions. On the other hand, the normalized viral load was lower in respiratory swab samples than in lesion swab samples, which was to be expected. Patients sought care at a more advanced stage of infection, in which lesions are already present in different phases.

In this study, virus was easily recovered in standard cell culture (VeroE6, MRC-5) from samples with a real-time qPCR cycle threshold of <31 and ≈3.3 log_10_ copies/10^3^ cells, according to a standard curve (*6*). Cytopathic effect appeared in <5 days. In addition, subcultures were achieved in other cell lines commonly used in the laboratory (A549 or LLC-MK2). Those data indicate that at higher viral loads, the virus is complete and transmissible, as has been demonstrated with other viruses such as SARS-CoV-2 (*11*). A limitation of this study was the lack of detailed clinical information for many patients.

In summary, MPXV requires rapid diagnosis and a rapid public health response. The designed real-time qRT-PCR and virus characterization proved very useful in diagnosing mpox and surveillance for MPXV and could aid in controlling the spread of infection and managing outbreaks. Furthermore, the use of culture can help confirm transmission. 
